# Glial Biologist's Guide to Mass Spectrometry‐Based Lipidomics: A Tutorial From Sample Preparation to Data Analysis

**DOI:** 10.1002/glia.24665

**Published:** 2025-01-03

**Authors:** Caitlin E. Randolph, Katherine A. Walker, Ruilin Yu, Connor Beveridge, Palak Manchanda, Gaurav Chopra

**Affiliations:** ^1^ Department of Chemistry Purdue University West Lafayette Indiana USA; ^2^ Department of Computer Science (By Courtesy) Purdue University West Lafayette Indiana USA; ^3^ Purdue Institute for Drug Discovery West Lafayette Indiana USA; ^4^ Purdue Institute for Integrative Neuroscience West Lafayette Indiana USA; ^5^ Purdue Institute of Inflammation Immunology and Infectious Disease West Lafayette Indiana USA; ^6^ Purdue Institute for Cancer Research West Lafayette Indiana USA; ^7^ Regenstrief Center for Healthcare Engineering West Lafayette Indiana USA

**Keywords:** glial lipidomics, lipid identification, lipidomics data analysis, lipids in neurological disease, mass spectrometry‐based lipidomics, sample preparation

## Abstract

Neurological diseases are associated with disruptions in the brain lipidome that are becoming central to disease pathogenesis. Traditionally perceived as static structural support in membranes, lipids are now known to be actively involved in cellular signaling, energy metabolism, and other cellular activities involving membrane curvature, fluidity, fusion or fission. Glia are critical in the development, health, and function of the brain, and glial regulation plays a major role in disease. The major pathways of glial dysregulation related to function are associated with downstream products of metabolism including lipids. Taking advantage of significant innovations and technical advancements in instrumentation, lipidomics has emerged as a popular omics discipline, serving as the prevailing approach to comprehensively define metabolic alterations associated with organismal development, damage or disease. A key technological platform for lipidomics studies is mass spectrometry (MS), as it affords large‐scale profiling of complex biological samples. However, as MS‐based techniques are often refined and advanced, the relative comfort level among biologists with this instrumentation has not followed suit. In this review, we aim to highlight the importance of the study of glial lipids and to provide a concise record of best practices and steps for MS‐based lipidomics. Specifically, we outline procedures for glia lipidomics workflows ranging from sample collection and extraction to mass spectrometric analysis to data interpretation. To ensure these approaches are more accessible, this tutorial aims to familiarize glia biologists with sample handling and analysis techniques for MS‐based lipidomics, and to guide non‐experts toward generating high quality lipidomics data.

## Graphical abstract

Introductory tutorial for glial biologists on mass spectrometry‐based lipidomics, outlining best practices, guidelines, and practical considerations for sample collection, preparation, lipid extraction, data acquisition, analysis, and interpretation.

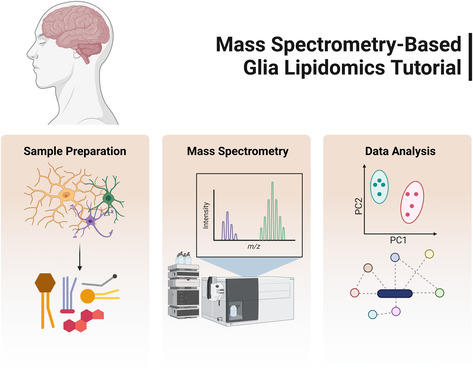

## 1 Introduction

Originally thought of as structural support to neurons in the nervous system, glial cells are now considered central to a diverse array of functions in development and diseases. Glial cells display a high degree of phenotypic and functional heterogeneity throughout life, an observation that has been increasingly appreciated with the rise of bulk and single‐cell omics approaches in combination with novel transgenic and cellular models. Consequently, glial cells are now known to play active, essential roles in brain development and function, including neurotransmission, metabolic support, neuronal insulation, and the elimination of pathogens, cellular waste, and debris. Given their highly diverse and specialized roles, glial cell dysfunction is linked to a broad range of neurological disorders, including developmental, psychiatric, neurotraumatic, and neurodegenerative diseases (Di Benedetto and Rupprecht 2013; Kim, Choi, and Yoon 2020; Kruyer, Kalivas, and Scofield 2023; Shao et al. 2022; St‐Pierre et al. 2022).

To better understand the roles of glia in the central nervous system, omics‐based approaches have quickly emerged as the frontrunners in analytical strategies to discover new targets. Global assessments of proteins, RNA, genes, metabolites, and lipids are correlated to proteomics, transcriptomics, genomics, metabolomics, and lipidomics, respectively. Although, by definition, the metabolome is comprised of a variety of biomolecules including lipids, amino acids, sugars, and organic acids, modern metabolomics predominantly focuses on the hydrophilic classes, while lipidomics has emerged as a standalone omics‐based discipline devoted exclusively to the complexities of organismal lipidomes (Wang et al. 2020).

**FIGURE 1:**
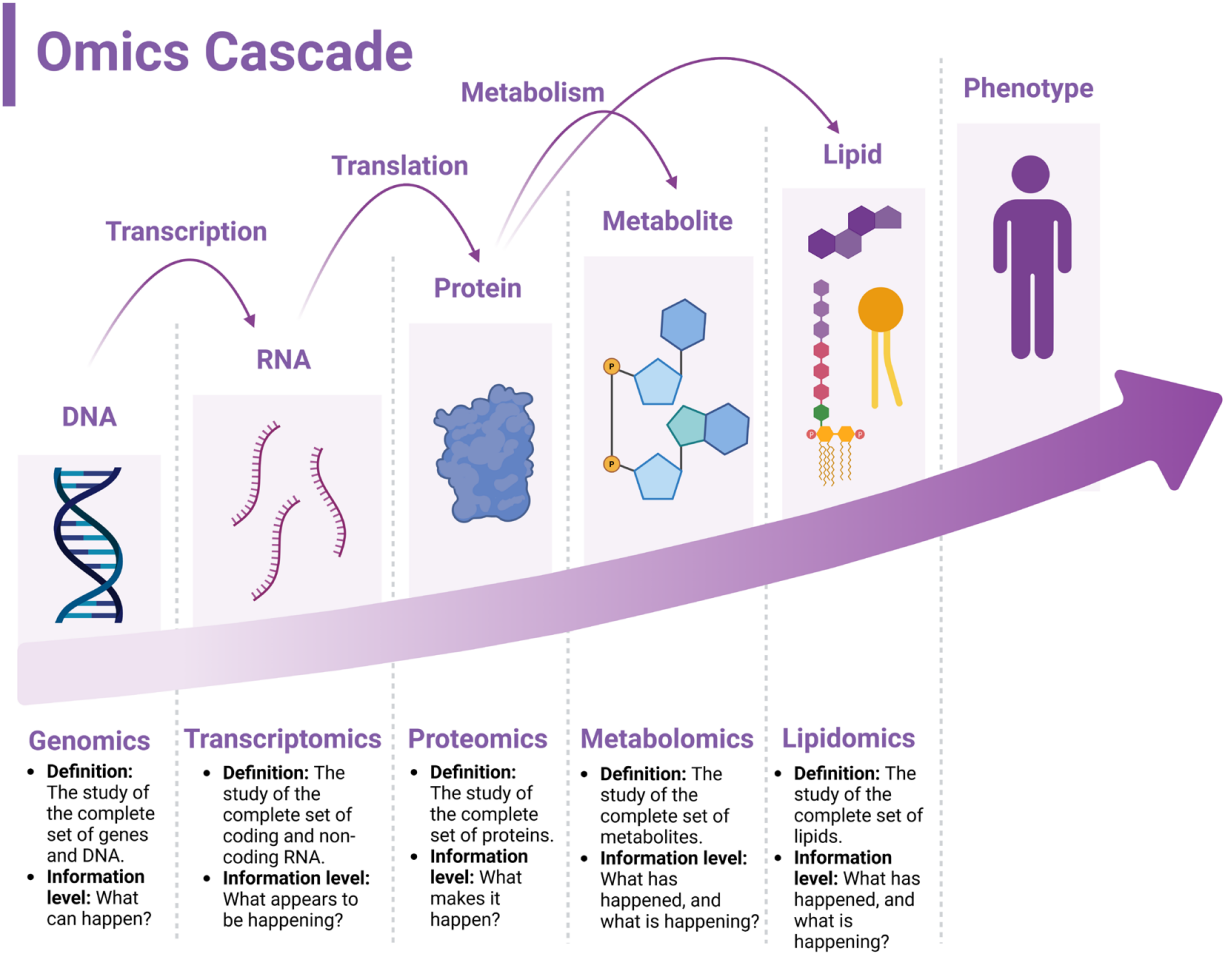
Schematic showing hierarchical transmission of information in biological systems through the omics cascade, encompassing several fields such as genomics, transcriptomics, proteomics, metabolomics, and lipidomics.

Figure 1 depicts the hierarchical information flow through biological systems via the omics cascade for the most popular “omics” disciplines from genes to metabolites and lipids. Although genomics, transcriptomics, and proteomics have a rich history of unraveling insights into cellular biology, metabolomics—and more recently lipidomics—offer significant advantages over other omics disciplines. Specifically, compared to the pace of transcriptome and proteome variations, the cellular lipidome fluctuates rapidly (i.e., millisecond to second timescale) in response to both cellular activity and environmental changes, offering a real‐time, direct readout of the biochemical processes and cellular state (Mika, Sledzinski, and Stepnowski 2018). These cellular states are related to cellular function and its response in a given environment when compared to alternate omics approaches. Additionally, lipids are highly conserved among living organisms, enabling streamlined analytical workflows across various sample types and origins. Together, lipidomics allows for the precise characterization of biological states for unknown phenotypes along with metabolic changes associated with known disease pathologies. Moreover, a growing body of evidence supports the utilization of lipid molecular structures as biomarkers for a diverse range of pathological, biological, and metabolic conditions, including neurodegenerative diseases like AD and Parkinson's disease (PD) (Poljak et al. 2020; Tokuoka et al. 2019; Casas‐Fernández et al. 2022; González‐Domínguez, García‐Barrera, and Gómez‐Ariza 2014; Sarkar et al. 2022; Wolrab et al. 2022; Jiang et al. 2021). To this end, we defined the scope of this review tutorial to focus on glia lipidomics, as we see great potential for continued future exploration of the glial lipidome to unravel novel pathways and treatments for several neurological and neurodegenerative diseases.

Typically, lipidomic analysis can be broadly classified into nontargeted and targeted approaches with specific benefits and drawbacks. Briefly, the goal of untargeted lipidomics is to comprehensively analyze every metabolite that may be found in a sample, including chemically unknown molecules. In general, untargeted approaches serve as a screening method to globally profile cellular lipid and metabolite landscapes and can achieve both qualitative identification and relative quantitation of lipid molecular species. Often, these untargeted screening approaches seek to measure the differences between two or more groups or biological situations (e.g., samples and controls) by detecting alterations in lipid profiles associated with a particular biological disturbance. Consequently, such approaches are used for hypothesis‐generation and are frequently used in conjunction with a pathway analysis to comprehend the functional implications of lipid alterations. These hypotheses can then be tested using additional analyses (such as targeted lipidomics) on a smaller cohort of samples. In short, targeted lipidomics measures specific groups of lipid species and are hypothesis driven. Targeted approaches are conducted when putative lipid identifications have been made, and further structural validation and/or absolute quantitation are desired. For example, targeted lipidomics generally focuses on the accurate quantification of specific lipid targets involved in relevant biological pathways (Xu et al. 2020). Additional references regarding experimental design, goals, and best practices for targeted versus untargeted workflows are provided here (Xu et al. 2020; Lippa et al. 2022; Lee and Yokomizo 2018; Cajka and Fiehn 2016). Importantly, both approaches require the incorporation and utilization of isotopically labeled standards. However, most untargeted methods rely on class‐specific internal standards to account for discrepancies in experimental protocols like lipid extraction or ionization efficiency, whereas targeted workflows should ideally employ an isotopically labeled standard for each individual lipid target to be quantified—though this is not always practical due to limitations on commercially available lipid standards. In other words, untargeted lipidomics studies use the internal standards mainly for monitoring lipid extraction recovery and quality control or analysis robustness rather than for quantification (Cajka and Fiehn 2016).

Lipid structures can be broadly classified based on their chemical structure and biological function into the following categories: fatty acyls, glycerolipids, glycerophospholipids (GPLs), sphingolipids, sterols, prenols, saccharolipids, and polyketides (Liebisch et al. 2020). However, the lipidome is highly diverse and consists of potentially thousands of distinct molecular species (Quehenberger et al. 2010). Such structural complexities arise from a vast number of potential acyl chain combinations and modifications, head group variations, and stereochemical configurations, to name a few. Further complicating lipidome analysis, lipid concentrations can span up to eight orders of magnitude (Ryan and Reid 2016). In turn, lipidomic investigations present significant challenges and demand analytical strategies with high specificity and sensitivity.

Due to its unparalleled sensitivity, selectivity, and versatility, mass spectrometry (MS) has become the tool of choice for modern lipidomics workflows (Randolph, Blanksby, and McLuckey 2020). Recent advances in MS‐based approaches have enabled the comprehensive profiling of thousands of lipids from tissue, fluid, or cell samples within a single experiment. The first step in any MS experiment involves sample ionization. Known as a soft‐ionization method, electrospray ionization (ESI) is the popular choice for lipid analysis, facilitating the efficient ionization of a broad range of intact molecular structures with little to no in‐source fragmentation of analytes. Lipid extracts can be directly introduced into the ESI source of a mass spectrometer either with or without prior chromatographic separation. Liquid chromatography‐MS (LC–MS) techniques are extensively employed in lipidomics to separate, identify, and measure complex mixtures of lipids. LC–MS combines the separation capabilities of LC with the sensitive and specific detection of MS, enabling detailed analysis of lipid species. Shotgun lipidomics does not rely on prior chromatographic separation and directly infuses a lipid extract into the mass spectrometer. This method provides a comprehensive and rapid analysis of lipid species in complex biological matrices, often in a high‐throughput manner. As the final step in an MS experiment, ions are separated by their mass‐to‐charge ratio (*m*/*z*) with relative resolution based on mass analyzer, and the results are recorded as a mass spectrum.

To date, few reports directly examine the glia lipidome, with most studies utilizing MS imaging to visualize cellular lipid distributions (Neumann, Ellis, et al. 2019; Hunter et al. 2018; Pereiro et al. 2020; Neumann, Comi, et al. 2019; Prakash et al. 2023; Guttenplan et al. 2021; Blank, Enzlein, and Hopf 2022). Astrocytes and oligodendrocytes are known to be relatively enriched with cholesterol, whereas microglia contain higher levels of sphingolipids (Fitzner et al. 2020). Because the lipidome signatures of cell types are not as well established as gene expression signatures, correlation between lipids and cells typically requires additional transcriptome analysis, imaging data, or cell purification prior to lipidomic analysis. Our group has pioneered glia global lipid profiling, as described in detail later (Prakash et al. 2023; Guttenplan et al. 2021; Beveridge et al. 2024). Consequently, the primary aim of this review is to encourage other glial biologists to familiarize themselves with the necessary workflows and protocols to probe glia lipidome alterations, primarily as a function of disease state in order to better understand neurological and neurodegenerative disease pathogenesis and treatments.

On the other hand, there is a rich history of lipid profiling to provide insight into neurological disease, as neurological disorders are associated with disruptions in the brain lipidome, some of which may be central to the pathogenesis of those diseases (Yoon et al. 2022). For instance, sphingolipid species such as ceramide and sphingosine‐1‐phosphate (S1P) are significantly altered in AD‐derived brain tissue, cerebral spinal fluid, and plasma (Haughey et al. 2010; He et al. 2010; Le Stunff et al. 2019). In a recent integrated omics study, the ceramide/sphingomyelin (SM) metabolism pathway is strongly associated with AD pathology based on the transcriptome, neuroimaging, and lipidomic data from ~1500 individuals (Baloni et al. 2022). Treatment with an S1P analog effectively rescued the cognitive deficit in 9‐month‐old APP/PS1 mice, although the exact mechanism remains unclear. Cholesterol is another important lipid class strongly implicated in neurodegenerative diseases. While astrocytes are considered to be responsible for the bulk of cholesterol production in healthy adult brains, evidence also suggests the compensatory participation of microglia or oligodendrocytes in cholesterol synthesis in disease/injury, or during development (Berghoff et al. 2021; Qian et al. 2022; Berghoff, Spieth, and Saher 2022). Neurons rely heavily on glial‐derived cholesterol transported via apolipoproteins APOE and APOJ. Lipidomic analysis of human brain tissue has revealed a disruption in levels of sterols and sphingolipids in AD patients with the *APOE4* genotype, but not in healthy controls (Yang et al. 2023; Bandaru et al. 2009). In aged *APOE* mice, global lipidomics also identified 35 significantly changed lipid species in the entorhinal cortex. The levels of several diacylglycerol, cholesterol‐ester, and sphingolipids were decreased, while the levels of ceramides and hexosylceramides were increased in *APOE4* mice compared to *APOE3* mice. While introducing significant lipidomic perturbations using genetic manipulations can be problematic, often due to embryonic lethality or the existence of highly redundant lipid metabolic pathways, global lipidomics analysis can offer critical insight into the remarkably dynamic and delicate changes in the lipidome of otherwise “physiologically normal” model organisms. While these reports do not directly investigate perturbations in glia lipid profiles, they demonstrate the overall utility of lipidomic profiling in neurological conditions.

**FIGURE 2:**
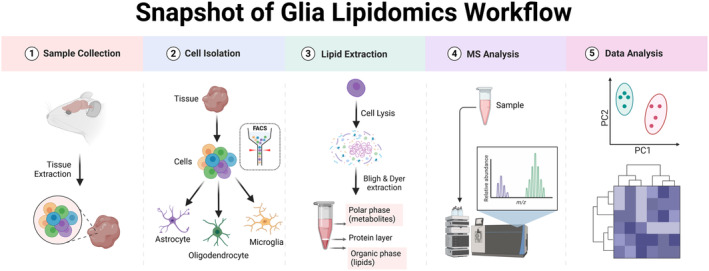
Overview of generic glia lipidomics workflow. The workflow includes sample collection, cell isolation, lipid extraction, MS analysis, and data analysis.

In this work, we present a tutorial guide for glia lipidomics, covering in a stepwise manner crucial aspects of analysis ranging from sample collection and preparation to MS workflows and analysis (Figure 2). Our aim is to familiarize glial biologists with the necessary components of MS‐based lipidomics approaches to ensure proper data collection and interpretation. We cover the basic principles of MS lipid analysis, calling attention to particular MS workflows and configurations required to facilitate various levels of lipid identification and quantitation. While mass spectrometry imaging (MSI) can also provide spatially resolved lipid identification and quantification within tissues, cells, and other biological samples, we focus the review on traditional MS approaches, as these are the most widely accessible. Our tutorial aims to provide an up‐to‐date, concise guide of MS‐based lipidomics to lead independent lipidomic investigations to explore glia biology. For reference, best practices and common pitfalls along with ways to avoid these errors in MS‐based lipidomics have been extensively reviewed elsewhere (Giles et al. 2018; Köfeler et al. 2021; Skotland et al. 2024; Wood and Cebak 2018; Liebisch et al. 2019). Finally, we strongly encourage glial biologists interested in MS‐based lipidomics to stay current on lipidomics standardization efforts, including reporting and experimental procedure, as outlined by the Lipidomics Standard Initiative (https://lipidomicstandards.org/) (Liebisch et al. 2019).

## 2 Sample Collection and Preparation

Modern protocols typically employ affinity‐based techniques like magnetic‐ or fluorescence‐activated cell sorting dependent on the presence of markers specific to each cell. This is performed after gentle mechanical or enzymatic dissociation of brain tissue. The purity of isolated cells can be verified using immunocytochemistry or flow cytometry for cell‐specific markers. Glial cell isolation from adult brains suffers from lower yields, and measures are needed to prevent cell activation while maintaining viability, making it comparatively more challenging than isolation from neonatal or embryonic brains. Isolated glia may be used immediately or plated for further manipulation prior to lipid/metabolite extraction. Alternatively, pure and stable glial cultures may be derived from induced pluripotent stem cells, which can preserve patient‐specific phenotypes (De Vries and Boullerne 2010; Li and Shi 2020; Santos, Mei, and Marchetto 2022; Clayton et al. 2024; Fattorelli et al. 2021).

Special considerations should be taken when moving on to the lipid extraction step(s). Cell culture media often includes growth factors like granulocyte macrophage colony stimulating factors (CSFs) and macrophage CSFs to support glial cell growth and proliferation. Buffers (e.g., PBS, HEPES) and small molecules (e.g., glucose, glutamine), that are used to support glial cell integrity may contaminate the final preparation and contribute to confounding matrix effects in analysis. The lipid extraction process in organic solvents naturally removes many of the buffer components and small polar molecules, resulting in a cleaner lipid fraction. Consequently, extracted lipids are usually resistant to such contamination. However, high concentrations of salts or amino acids from buffers or media can suppress the ionization of less abundant metabolites that are found in the aqueous phase during separation making their detection challenging.

Some precautionary measures and considerations can certainly improve detection and reduce matrix effects for MS lipidomics. For example, repeated washing of cells before extraction can get rid of any residual media components. Internal standards labeled with stable isotopes can be used to distinguish between endogenous and foreign compounds. Utilization of more selective extraction methods and additional purification steps are especially effective for the enrichment of aqueous metabolites. For example, hydrophilic interaction LC‐solid‐phase extraction (SPE) can be used to selectively retain polar metabolites while mixed‐mode ion exchange SPE can separate metabolites according to their charge and polarity (Mushtaq et al. 2014; Alsaleh et al. 2019). The components of the isolation and culture media must be carefully chosen in order to minimize interference with the target metabolites. When focusing on metabolite analysis, serum‐free media are frequently chosen for glial cell cultures since serum contains varying quantities of growth factors, hormones, vitamins, and proteins which may cause problems in the identification of metabolites. Further, media should not contain any compounds that can potentially activate glia, especially microglia, or mimic inflammatory signals. The concentration of glucose in the media can also substantially impact cellular metabolism, thus for lipidomics studies, lower glucose concentrations are often used to reduce interference and better reflect physiological conditions (Orihuela, McPherson, and Harry 2016). Further, instead of l‐glutamine, using stabilized forms of glutamine (e.g., GlutaMAX), can reduce degradation products like ammonia and pyroglutamate which negatively impact cell viability (Wei et al. 2022). Thus, it is imperative to carefully select isolation and culture media components in order to minimize interference with the lipids of interest. Finally, short‐term culturing cells in metabolite‐free medium prior to cell harvesting can be beneficial in minimizing contamination.

When collecting glia from culture experiments, the cells will also need to be detached. This can be accomplished using trypsin and/or mechanical detachment with scraping. Astrocytes readily detach from cell culture flasks, but microglia may require multiple methods of detachment to retrieve most cells. Cells adapt quickly to changes in their environment; this means that metabolic changes can occur in seconds. To preserve the metabolic state in glia prior to detachment, a quenching step may be added. Quenching is done by rapidly cooling the cells though the application of a cold quenching solvent such as liquid nitrogen, a methanol/water mixture, or saline directly to the cells (Wang et al. 2023; Hu and Zhang 2018). To prevent metabolite changes brought on by post‐mortem enzymatic processes, tissue/cell collection should be done as quickly as possible and immediately frozen after collection (Dudzik et al. 2018). The optimal quenching technique can affect metabolite recovery, and thus should be chosen based on the particular cell type and metabolites of interest. It is crucial to validate quenching procedures by evaluating the stability of essential metabolites under the selected conditions and by utilizing stable isotope‐labeled internal standards.

## 3 Lipid Extraction

Following glia isolation, cells must be lysed before or during lipid extraction. Often, cell lysis can be achieved during the process of lipid extraction via the addition of organic solvents. However, a variety of cell lysis methods have also been developed independent of lipid extraction and are largely dependent on factors like cell type and downstream applications (Shehadul Islam, Aryasomayajula, and Selvaganapathy 2017). It is important to note that choosing the right lysis method requires considering the balance between effective cell destruction and maintaining the integrity of the target molecules for subsequent analysis. Moreover, decoupling the lysis and lipid extraction steps can reduce quantitation accuracy due to differences in extraction efficiency—a notion that must be thoughtfully considered if quantitation is a goal. Furthermore, to account for disparities in extraction efficiency, it is imperative that internal standards be introduced as early as possible during sample preparation (i.e., prior to cell lysis or lipid extraction). These standards should be similar to the target lipids but distinct enough to be resolved by MS. Importantly by adding these standards at *known* concentrations before the extraction process, you can correct for losses or variations that occur during extraction, sample preparation, or analysis, ensuring the most accurate results. The best practice is to opt for isotopically labeled lipid standards, employing at least one internal standard per lipid class (or subclass) you aim to analyze. Finally, choosing the appropriate internal standard concentration is important, particularly if quantitative analysis is required. Specifically, internal standard concentrations should fall within the range of endogenous lipid concentrations, considering overly high concentrations of internal standards can lead to ion suppression and reduced sensitivity for native lipids, which can all negatively impact lipidomics data quality.

In general, glial cell lysis can be achieved through a broad range of mechanical, chemical, enzymatic, and temperature‐dependent processes. However, not all cellular lysis techniques are appropriate to use in lipidomic investigations. For example, though sonication can effectively lyse glia, sonication can produce excessive heat and can lead to lipid degradation, posing a threat to the lipidome integrity. Similarly, chemical‐based lysis procedures should be used with necessary precautions to preserve the lipidome. First, all reagents used for cell lysis and lipid extraction should be of high quality and at a minimum HPLC‐grade, though ultrapure solvents are preferred. The usage of subpar reagents and solvents can lead to poor MS performance and data quality and should therefore be avoided. Second, detergents (e.g., sodium dodecyl sulfate or Triton X‐100), strong alkaline or acidic conditions, and oxidizing reagents (e.g., hydrogen peroxide) are not recommended. Utilizing the aforementioned conditions for cell lysis could lead to lipid degradation or modification, giving rise to inaccurate reflections of cellular lipid content and composition. It is also strongly advised that detergents be avoided in MS‐based lipidomics, as detergents can significantly interfere with MS analysis by causing ion suppression, adduct formation, instrument contamination, and complex data interpretation. To mitigate these issues, it is crucial to use detergent‐free lysis methods and, when possible, remove detergents before MS analysis, or use MS‐compatible detergents with extreme caution. Proper sample preparation and cleanup are essential to ensure accurate and precise MS results. For MS‐based glia lipidomics, we recommend utilizing mild lysis buffers void of detergents and harsh pH conditions. Ideally, lysis should be conducted at low temperatures (e.g., on ice) and in an inert atmosphere (e.g., nitrogen) to mitigate lipid degradation and oxidation, respectively. Although inert atmospheric conditions are not always feasible, lysis should be achieved as quickly as possible to preserve lipidome integrity.

After lysis, centrifugation can be employed to pellet cell debris. We note that the impact of centrifugation on lipidomics experiments depends on the specific step in which it is applied and the conditions used. For example, employing centrifugation after cellular lysis but before lipid extraction can impact lipidomics data, as this approach will likely result in the loss of most organellar lipids—a consequence that can be exploited intentionally. In brief, the pre‐extraction centrifugation step is typically used to remove large cellular debris, unbroken cells, or unwanted fractions (like subcellular organelles) from a sample. It is therefore important to note and accurately report sample preparation steps, as the given order of sample preparation steps can potentially induce positive, negative, and even unintended effects on the lipidomic profile.

Next, lipids can be extracted from cell supernatant or fractionated, intact cellular samples. Choosing the right lipid extraction method depends on the specific lipid classes of interest, sample type, and downstream application (Cajka and Fiehn 2014; Li et al. 2014; Ulmer et al. 2018). For example, untargeted lipidomics necessitates the utilization of non‐discriminatory extraction procedures that can extract all detectable lipid classes in a sample, regardless of their concentration, while minimizing contamination from non‐lipid components. On the other hand, for targeted lipidomics applications where absolute quantitation of specific lipid species or classes is often desired, it is crucial to use lipid extraction methods that provide high recovery, reproducibility, and minimal interference for target lipid species. In both targeted and untargeted lipidomics workflows, liquid–liquid protocols have proven highly effective (Saini et al. 2021; Wong et al. 2019). Liquid–liquid extraction protocols offer the major advantages of broad lipid class coverage and high recovery. These methods are also straightforward and easy to implement, lending themselves to high‐throughput workflows. SPE is also a useful approach in lipidomics for extracting lipids. It is employed for sample cleanup, fractionation, and enrichment of particular lipid classes. For these reasons, SPE is ideal for targeted lipidomics. However, SPE can be time‐consuming, require method optimization, and can be more expensive due to the need for specialized SPE cartridges and sorbents. We note that in some cases, a combination of liquid–liquid extractions and SPE could be utilized to leverage the strengths of both techniques and provide the best results.

As liquid–liquid extraction protocols can be universally applied to both targeted and untargeted lipidomics studies, liquid–liquid extraction methods serve as a critical step in glia lipidomics workflows. Table 1 outlines some popular liquid–liquid lipid extraction protocols that are amendable for glial lipidome investigations. Detailed reviews regarding lipid extraction protocols can be found elsewhere (Saini et al. 2021; Aldana, Romero‐Otero, and Cala 2020). It is important to note that no single extraction technique can extract all known lipids of interest. For example, hexane‐based extraction protocols are best suited for nonpolar lipid applications (i.e., triacylglycerols [TGs], cholesterol esters, etc.), as more polar lipids like GPLs are not efficiently extracted. In other words, careful consideration and optimization are required when choosing a lipid extraction method, as variations in extraction can influence the recovery and detectability of certain lipid classes. Furthermore, it is crucial to consider inherent biases introduced by extraction methods due to differences in solvent polarity, phase partitioning, and extraction efficiency to obtain accurate and representative lipidomics data. In general, when choosing an extraction method, first consider the lipid classes of interest that ultimately aligns with maximizing recovery for those lipid species. Additionally, it is important to note that sequential extractions or combinations of extraction methods can be utilized, especially if a comprehensive lipid profile is required.

**TABLE 1:** Summary of common lipid extraction protocols.

Method	Solvents	Solvent ratio	Notes
Folch, Lees, and Stanley (1957)	Chloroform:methanol	2:1	High yield, high reproducibility, broad lipid coverage, toxic solvents (chloroform)
Bligh and Dyer (1959)	Chloroform:methanol:water	2:2:1.8	High yield, high reproducibility, broad lipid coverage, toxic solvents (chloroform)
Matyash et al. (2008) (MTBE)	Methyl‐tert‐butyl‐ether:methanol:water	10:3:2.5	High yield, broad lipid coverage, use of green solvents, possible ion suppression from increased sodium adducts
Butanol/methanol (BUME) (Cruz et al. 2016; Löfgren, Forsberg, and Ståhlman 2016; Löfgren et al. 2012)	Butanol:methanol	3:1	High yield, broad lipid coverage, single‐phase, amenable to automatization, use of green solvents
Hara and Radin (1978)	Hexane:isopropanol	3:2	Nonpolar to moderately polar lipids, use of green solvents, incomplete extraction of highly polar lipids

The general steps for liquid–liquid extraction of lipids from cell lysate are as follows: (1) solvent addition, (2) mixing, (3) phase separation, (4) centrifugation, (5) organic phase collection, (6) evaporation, and (7) reconstitution. In most cases, biphasic solutions are obtained by the addition of water or an alternative phase‐inducing reagent and subsequent centrifugation. Due to their hydrophobic nature, lipids will accumulate in the organic layer of the biphasic solution. Following the careful collection of the organic layer, this lipid‐containing layer can be dried down using a rotary evaporator or a stream of nitrogen. Evaporated lipid extracts can be reconstituted in a desired volume of chosen organic solvent and diluted appropriately prior to MS analysis. Proper optimization of lipid extraction protocols, including the use of internal standards and cold extraction, are encouraged to ensure the best results.

## 4 MS Analysis

### 4.1 Sample Ionization—ESI

Mass spectrometry has emerged as the tool of choice for modern lipidomics (Ryan and Reid 2016; Hu and Zhang 2018; Blanksby and Mitchell 2010; Randolph et al. 2023). Although alternative soft‐ionization methods like matrix‐assisted laser desorption ionization and desorption ESI have been mainstays of mass spectrometric imaging approaches for spatial lipidomics, ESI‐MS‐based lipidomics can be implemented in a variety of ways (i.e., shotgun, LC‐based, targeted, nontargeted, etc.), offering a highly versatile and flexible platform for lipidomic analysis. Consequently, ESI‐MS is by far the most popular and widely accessible mass spectrometric technique. Here, we present introductory information, guidelines, and general recommendations for glia lipidomics exclusively focused on ESI‐MS approaches.

As a first step in any MS‐based lipidomics experiment, the lipid extract is directly infused into the ESI source, where ionization of lipid molecular species occurs. Both understanding and recognizing patterns in possible ion types for individual lipid analytes are integral for accurate lipid annotation and identification, enabling the formulation of robust conclusions. Without this knowledge, lipidomic data risks incomplete or incorrect interpretation, undermining experimental results and biological significance. To aid in proper data annotation, popular ionization strategies for common lipid classes and subclasses are presented in Table 2. In general, lipids can be ionized in either positive or negative ion modes, depending on their chemical functionality and polarity (Randolph, Blanksby, and McLuckey 2020; Pulfer and Murphy 2003). For example, acidic lipids like fatty acids (FAs), glycerophosphoethanolamines (PEs), glycerophosphoglycerols, glycerophosphoserines (PSs), glycerophosphoinositols (PIs), glycerophosphatidic acids, and cardiolipins form deprotonated ions of the form [M − H]^−^ with negative ion mode ESI. Lipid structures containing sulfate or hydroxyl groups such as sulfatides, gangliosides, and galactolipids can also be detected via negative ion mode ESI. Characterized by containing a choline group, ESI of glycerophosphocholines (PCs) and SM are detected predominately in positive ion mode due to their fixed cationic charge. In the absence of polar functional groups, neutral lipids like glycerolipids (e.g., TGs, diacylglycerols, monoacylglycerols) and sterol lipids (e.g., cholesterol and cholesterol esters), are reliant on, or their coverage and sensitivity are greatly enhanced, by exploiting chemical derivatization strategies for ionization via ESI. By doping formate or acetate‐based salts into a lipid extract, neutral lipids can be detected as ammonium, potassium, or sodium adduct cations. We also note that by doing so, most acidic lipids will also yield adduct ions via positive ESI, and in turn, the resulting mass spectra can be complicated by the generation of multiple lipid cationic species and data analysis should account for potential overlaps in associated *m*/*z* values.

**TABLE 2:** Ionization strategies for various lipid classes.

Lipid class	Lipid category	Positive ion mode	Negative ion mode
PC	Glycerophospholipids	[M + H]^+^, [M + Na]^+^, [M + K]^+^	[M + Cl]^−^, [M + HCOO]^−^, [M + CH3COO]^−^
PE		[M + H]^+^, [M + Na]^+^	[M − H]^−^
PA			[M − H]^−^
PS		[M + H]^+^	[M − H]^−^
PI		[M + NH_4_]^+^, [M + Na]^+^, [M + H]^+^	[M − H]^−^
PG		[M + NH_4_]^+^, [M + Na]^+^, [M + H]^+^	[M − H]^−^
Fatty acids	Fatty acyls		[M − H]^−^
Acylcarnitine		[M + H]^+^, [M + Na]^+^	
TG	Glycerolipids	[M + NH_4_]^+^, [M + Na]^+^	
DG		[M + NH_4_]^+^, [M + Na]^+^, [M − H_2_O + H]^+^	
MG		[M + NH_4_]^+^, [M + Na]^+^, [M − H_2_O + H]^+^	
Cholesterol	Sterols	[M − H_2_O + H]^+^, [M + NH_4_]^+^, [M + Na]^+^	
CE		[M + NH_4_]^+^, [M + Na]^+^, [M + H]^+^	
SM	Sphingolipids	[M + H]^+^	[M + HCOO]^−^, [M + CH_3_COO]^−^
Ceramides		[M + H]^+^	[M − H]^−^

### 4.2 Mass Analyzers

ESI is compatible with a wide range of mass analyzers. Mass analyzers are key components of mass spectrometers and are responsible for separating ions based on *m*/*z* ratio. Different types of mass analyzers have distinct benefits in terms of resolution, sensitivity, mass range, and speed. Traditional triple quadrupole (QqQ) instruments continue to have a prominent role in lipidomic investigations, particularly for quantitative analysis. QqQ instruments offer high sensitivity and fast scanning capabilities, making them suitable for both quantitative and qualitative analysis. However, QqQ platforms lack resolution compared to alternative high‐resolution analyzers like time‐of‐flight (TOF) or Fourier transform (FT)‐based instruments like Orbitrap and Fourier‐transform ion cyclotron resonance (FT‐ICR) mass spectrometers. Consequently, the use of high‐resolution MS (HRMS) has significantly enhanced the potential for examining lipid species (Züllig and Köfeler 2021). Table 3 compares common mass analyzer features for popular commercial platforms like quadrupole TOF (QTOF), linear ion trap, QqQ, Orbitrap, and FT‐ICR.

**TABLE 3:** Common mass analyzer features.

Mass analyzer	Typical mass resolution^a^	Typical mass accuracy	Limit of detection (LOD)
QqQ	1000	~100 ppm	Low femtomole to attomole
Orbitrap	~12,500–480,000	< 5 ppm	Low picomole to femtomole
FT‐ICR	1,000,000	< 1 ppm	Low femtomole to attomole
QTOF	10,000–100,000	2–5 ppm	Low picomole to femtomole
LIT	1000–3000	~100 ppm	Low picomole to femtomole

^a^ Resolution at full width half maximum (FWHM) (reported at *m*/*z* 200 for Orbitrap and FT‐ICR).

### 4.3 Full Scan MS

A full scan mass spectrum or MS1 scan is generated by detecting all ionized compounds within a specific *m*/*z* range and can be useful in untargeted approaches where broad coverage is required or can serve as an initial step in tandem‐MS (MS/MS) experiments. MS1 scans can be beneficial for general sample profiling as they capture a comprehensive view of the entire sample. From an MS1 spectrum of a lipid extract, putative lipid identifications can be made via an accurate mass measurement (i.e., observed *m*/*z* value) and database matching. However, the accuracy of these putative lipid identifications is directly dependent upon mass analyzer resolution. Explicitly, resolving power can aid in lipid isobar discrimination. In short, lipid isobars are lipids with the same nominal (integer) masses but different exact masses, indicating differences in lipid structure and identity. Consequently, high resolution MS is encouraged for any glia lipidomics investigations reliant on MS1 scans alone, though it is advised that additional tandem‐MS experiments or separation strategies (e.g., LC, ion mobility [IM]) should be utilized if available to aid in lipid identification.

### 4.4 Conventional Tandem MS

Tandem MS (MS/MS) relies on the fragmentation of lipid precursor ions via collision‐induced dissociation (CID) to yield product ions, generating what is referred to as an MS2, MS/MS, CID, or product ion scan. Observed fragmentation patterns can be exploited to enhance lipid identification and structural characterization. In the case of GPLs, class‐specific fragmentation pertaining to headgroup composition allows for the detection of individual GPL classes (Pulfer and Murphy 2003). For example, PCs and SM precursor cations both yield abundant phosphocholine head group product ions detected at *m*/*z* 184 which serve as a diagnostic indicator for the PC and SM subclasses. In negative ion mode, dissociation of PI lipid precursors generates diagnostic product ions at *m*/*z* 241 which correspond to an inositol phosphate structure characteristic of the PI subclass. To identify PS and PE lipids, fragmentation pathways reliant on the neutral loss (NL) of the GPL headgroup can be exploited. For instance, in the negative ion mode, fragmentation of PS lipid anions yields abundant product ions originating from the NL of the serine (NL 87 Da) or phosphoserine moieties (NL 185 Da). Similarly, an NL of the PE head group (NL 141 Da) in either positive or negative ion ESI‐MS/MS can be exploited for PE lipid screening. To obtain information regarding acyl chain composition, negative ion ESI‐MS/MS is advantageous for GPL structures. For example, acidic GPL anions along with chloride/acetate adducted PC anions will undergo fragmentation via ester bond cleavage at the *sn*‐1 and *sn*‐2 positions of the anionic GPL. Consequently, fatty acyl carboxylate anions are liberated, facilitating the assignment of fatty acyl composition. In a similar fashion, fatty acyl composition of glycerolipid structures (i.e., TG, DG, and MG) can be obtained via CID of their respective adduct cations. Here, the dissociation of sodium or ammonium adducted glycerolipid precursor ions results in the efficient formation of a series of product ions corresponding to the NLs of individual fatty acyl chain constituents. In short, MS/MS is advantageous in lipid isobar discrimination, particularly in the absence of HRMS, and can provide some degree of isomeric resolution. For instance, isomeric lipid structures with varying fatty acyl chain sum compositions, head‐group compositions, or, in some cases, acyl chain regiochemistry (i.e., relative position of acyl chains on the glycerol backbone referred to as *sn*‐position) can be identified utilizing MS/MS alone. However, we note that *sn*‐positional assignments should be made with caution when relying on conventional ESI‐MS/MS alone, as these experiments do not generate unique product ions that can be correlated with *sn*‐position and instead are contingent on calibration approaches with authentic reference standards or enhanced separation strategies (Fabritius and Yang 2023). Additional stages of CID, known as MS^*n*^, can aid in enhanced structural elucidation, but are accessible in only certain instrument types with trapping capabilities, including ion traps, Orbitraps, hybrid mass spectrometers (e.g., QTOF), and FT‐ICR mass spectrometers. Alternatively, it is important to note that HRMS or MS^3^ are required to effectively discern isomeric/isobaric ether GPL subclasses like plasmalogens from their diacyl GPL counterparts (Hsu and Turk 2007; Randolph, Shenault, et al. 2020; Hsu et al. 2014). As ether lipids have recently been tied to various neurodegenerative pathologies, glia lipidomics investigations can greatly benefit from the utilization of combinations of high‐resolution platforms, advanced separation strategies (e.g., LC, IM), and CID (Kelley 2022; Dakterzada et al. 2023).

**FIGURE 3:**
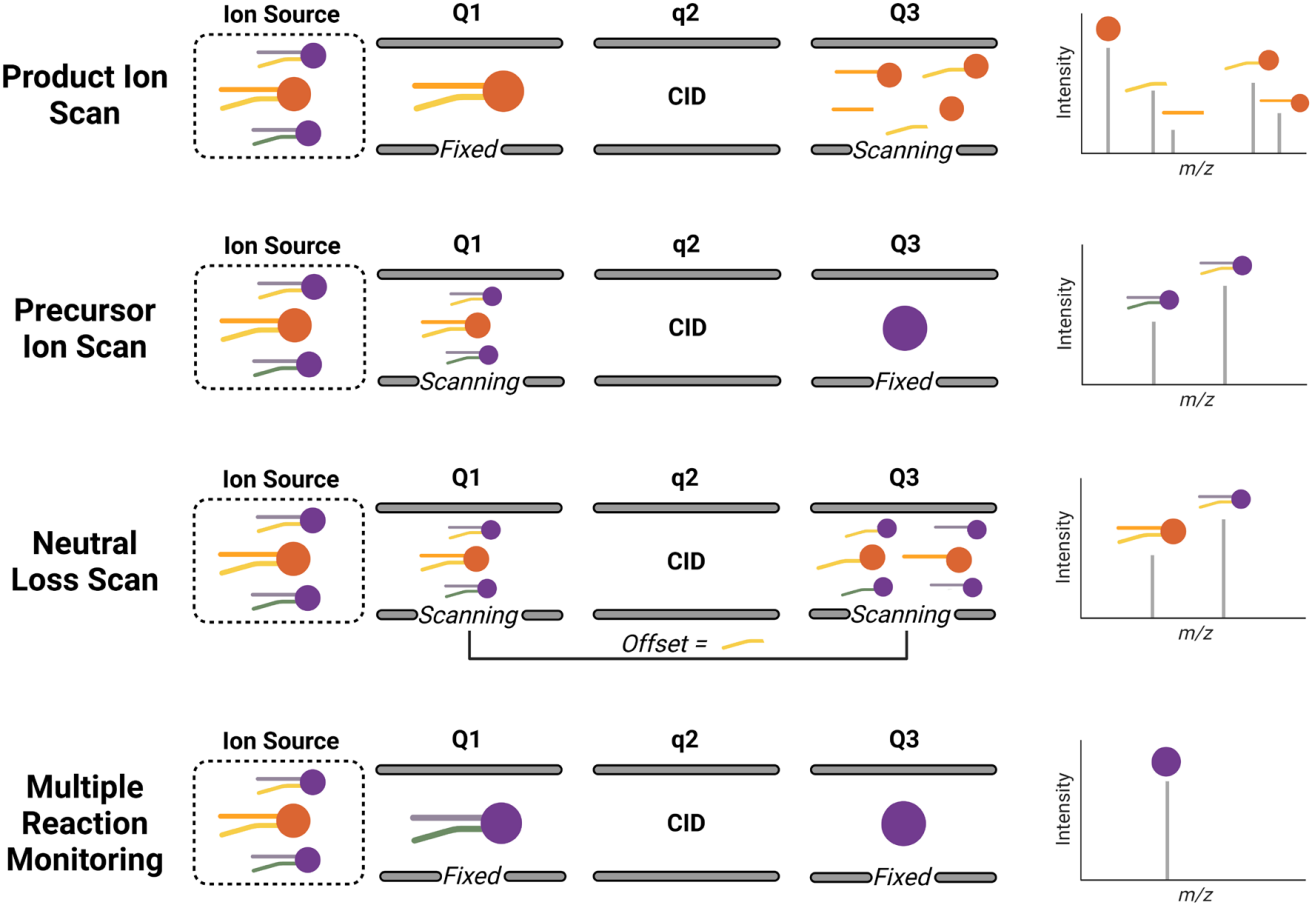
Schematic representation of various MS/MS scan types utilized in lipidomics.

To improve the speed and efficiency of lipid identification and quantitation, additional scan modalities dependent on MS/MS can be employed (scan types are summarized in Figure 3). In addition to a typical product ion scan (MS/MS), instruments like QqQ and hybrid quadrupole ion traps (QTRAP) mass spectrometers can conduct precursor ion, NL, and multiple reaction monitoring (MRM) scans. We note that while alternate configurations like Orbitrap or QTOF mass spectrometers systems cannot perform classic precursor ion scans (PISs) due to their design, they offer alternative approaches—such as data‐dependent acquisition, data independent acquisition (DIA), or targeted NL analysis—that can provide similar information for lipidomics and other molecular applications.

For all scan modalities, fragmentation occurs in the high‐pressure collision cell, q2. In a PIS, the first quadrupole mass filter (Q1) is scanned, while the third quadrupole mass filter (Q3) is fixed, operating as a mass selector for a specific *m*/*z* value. Here, the output represents all precursor ions values with the same *m*/*z* product ion. Alternatively, in an NL scan (NLS), both Q1 and Q3 are scanned simultaneously, but Q3 is offset by the NL under investigation. The output of a NLS reflects only the precursor ions that produce product ions generated via the indicated NL. Finally, MRM is the most sensitive scan type to date and can be adapted to nanogram‐scale samples. MRM profiling is capable of profiling lipid classes at the species level in which the lipid class, the sum of carbon atoms, the number of double bond equivalents, and additional oxygen atoms are reported. To perform an MRM‐scan, Q1 and Q3 are fixed. Explicitly, a desired lipid product ion is selected in Q1, fragmented in q2, and a targeted product ion is selected in Q3. The output reflects the intensity of the precursor/product ion transition pair.

In recent years, a shotgun approach (i.e., no chromatographic separation) referred to as MRM‐profiling emerged as a popular MS/MS technique for lipid screening (Edwards et al. 2021; Xie et al. 2021; Xie et al. 2019; Reis et al. 2023). MRM‐profiling is usually a two‐step process, where the first step is a discovery stage in which precursor ion and neutral loss scans are exploited to screen for functional groups associated with specific lipid classes. Data obtained from the discovery step can then be organized to generate a list of precursor and product ion transition pairs, referred to as MRMs, to profile individual samples for the designated lipid species. Alternatively, MRM‐profiling can be used in both the discovery and screening steps (Reis et al. 2023). MRM profiling offers the additional advantage of screening for both reported and undiscovered lipid structures, using simulated or predicted MRM transitions in conjunction with lipid databases and well‐understood fragmentation patterns of various lipid classes. Ultimately, the data generated from MRM profiling is intended as a screening step, serving as a guide to plan additional studies in which the structure of molecular species can be confirmed using alternative methods such as LC–MS/MS.

**FIGURE 4:**
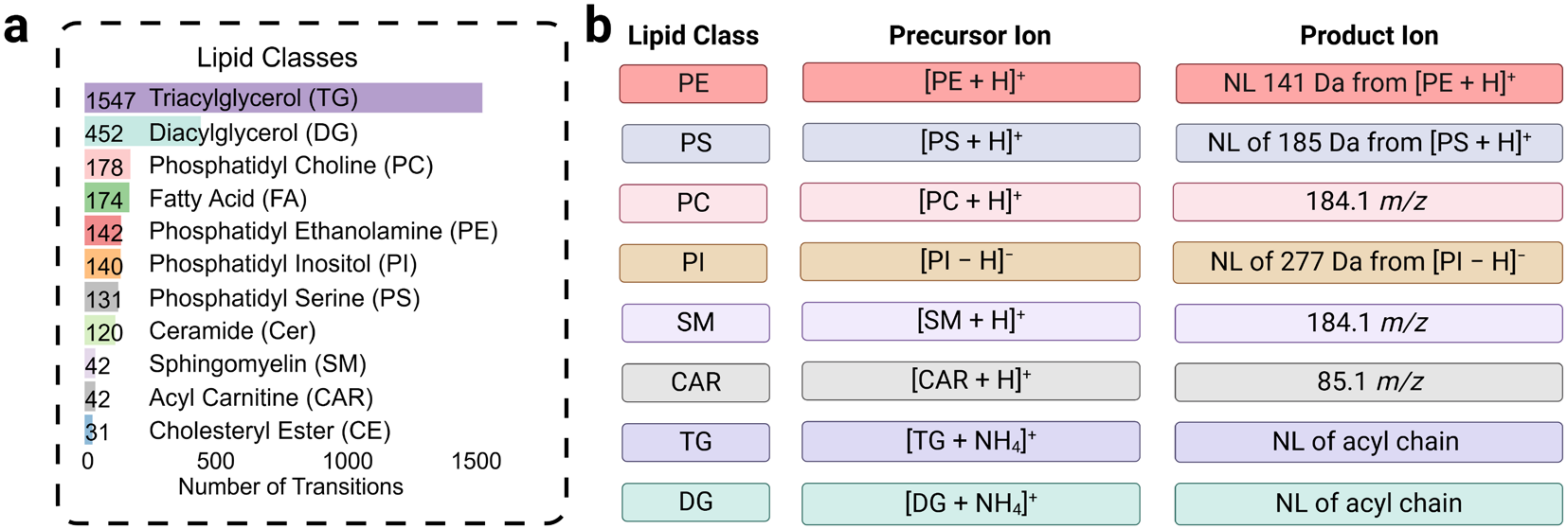
Summary of MRM profiling methods in the Chopra lab for global, unbiased glia lipidomics. (a) Distribution of MRM transitions by lipid class. (b) Common precursor and product ion transitions for several notable lipid classes.

To highlight the importance and successes of MRM‐based glia lipid profiling, several notable recent studies are summarized below. Explicitly, our group has pioneered comprehensive MRM profiling for glia lipidomics. To date, in‐house MRM methods enable the identification of nearly 3000 lipid species spanning 11 lipid classes (Figure 4). Applications of these workflows to identify specific lipid molecules in Alzheimer's disease (AD) pave the way for the targeting of novel glial mechanisms to combat neurodegeneration. Previously, we have shown that reactive astrocytes can release long‐chain saturated free FAs (FFAs) which are neurotoxic to injured neurons and oligodendrocytes (Guttenplan et al. 2021). Inhibition of the astrocytic fatty acyl enlongase ELOVL1 protected neurons and oligodendrocytes from astrocyte‐induced death. More recently, Prakash et al. have shown that Aβ is sufficient to induce lipid droplet (LD) formation in microglia and that this conversion requires a specific metabolic conversion of FFAs to TGs—the building blocks of LDs (Prakash et al. 2023). Chronic accumulation of these droplets leads to dysfunction in microglial phagocytosis of Aβ. Therefore, by chronically turning on a key pathway that catalyzes FFA conversion to TGs, microglia become less efficient phagocytes, and thereby microglia are less capable of clearing Aβ. In chronic neuroinflammation of AD, as the amyloid load becomes too much for microglia to clear, they likely switch from emergency responders to damage containers by reducing cytoplasmic FFAs load and converting them to TAGs for stable, long‐term energy storage. We have further contributed to our understanding of LD‐accumulated glia in aging and neurodegeneration by teaming up with the Bonini group. Here, Bryns et al. found that neuronal mitochondrial failure with aging can trigger the appearance of senescent glia in Drosophila (Byrns et al. 2024). Utilizing MRM‐profiling of Drosophila brains, we identified a lipid‐related mechanism by which senescent glia contributes to LD accumulation in non‐senescent glia during aging. This suggests that mitigating senescent glia may have a positive effect on improved health and that protecting neuronal mitochondrial function may be necessary for effective anti‐aging treatments. In a final example, MRM‐profiling was also employed to unravel lipid roles in spinal cord injury. Given the multi‐faceted role of microglial lipids and their interaction with reactive astrocytes, in collaboration with the Burda group, we found that lesion‐remote astrocytes regulate lipid metabolism of neighboring phagocytic microglia characterized by lipid accumulation (McCallum et al. 2024). Specifically, the loss of astrocytic *Ccn1* led to excessive, aberrant activation of local microglia in parallel with (i) abnormal molecular specification, (ii) dysfunctional myelin debris processing, and (iii) impaired lipid metabolism in LD accumulation in microglia, culminating in blunted debris clearance and attenuated neurological recovery from spinal cord injury. Importantly, MRM‐profiling provided lipid species identifications within these lipid‐accumulating and phagocytic‐impaired microglia. Additionally, McCallum et al. found that brain white matter degeneration‐associated microglia from injured astrocytic *Ccn1*−/− mice reduced LD formation. In combination with results from lipid MRM‐profiling, including lipid pathway analysis connecting lipid molecules to specific genes/proteins, we further identified increased cholesterol efflux pathway activated in these microglia (Saher et al. 2005). Together, these works specify the interplay between microglia and their environment suggesting a deeper relationship between glial lipids and disease pathology. Future work of deep profiling of different lipids in different brain regions in aging and neurodegeneration is underway.

### 4.5 Enhanced MS Strategies

**FIGURE 5:**
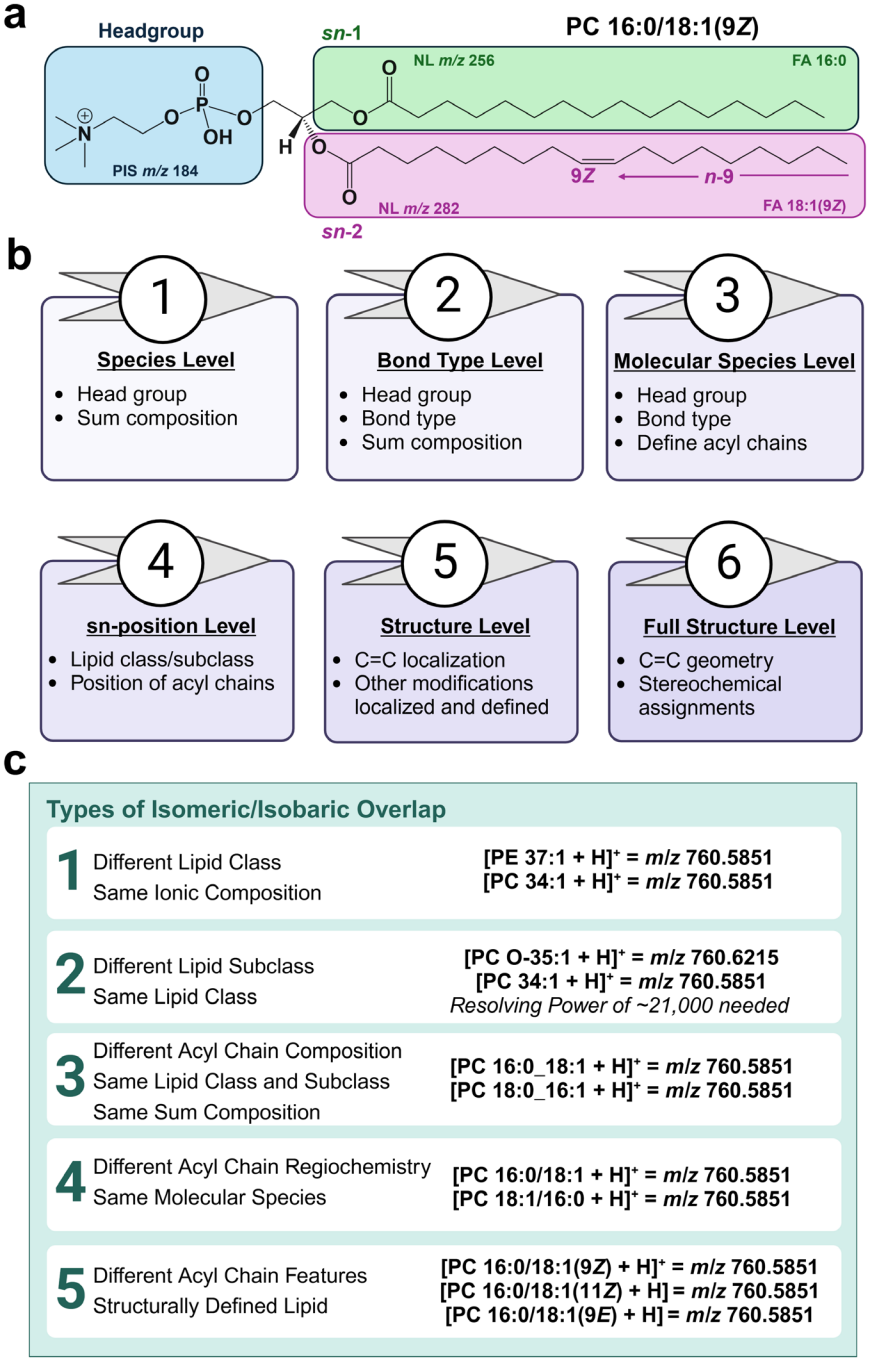
(a) Representative structure of PC 16:0/18:1(9Z). Headgroup and fatty acyl composition are identifiable via conventional MS/MS strategies, including the utilization of precursor ion and NLSs. For example, a PIS of *m*/*z* 184 identifies the PC headgroup in the positive ion mode, while NL scans of *m*/*z* 256 and 282 enable acyl chain composition assignments in the negative ion mode. However, additional structural features including acyl chain bond type and stereochemistry along with unsaturation site position and geometry require enhanced MS strategies. (b) Lipid structural identification hierarchy based on current classification and annotation recommendations. The features listed in each icon need are required to achieve a given level of lipid structural annotation. (c) Examples of common isomeric/isobaric overlaps for PC 34:1.

An overarching goal of lipidomics is to structurally define and quantitate all lipids in a biological system. While seemingly a rudimentary step in lipidomics, in‐depth lipid identification is confounded by an exorbitant number of distinct lipid structures, stemming from diverse lipid structural complexity. For example, as of today (July 2024), the LIPIDMAPS Structure Database contains nearly 49,000 unique lipid structures, spanning the 8 designated lipid classes. As the primary building blocks of lipid structures are largely limited to a handful of elements, many isomeric and isobaric *m*/*z* overlaps arise, representing a significant challenge for MS‐based lipidomics workflows (Figure 5c). While informative, CID alone is not capable of discerning all lipid structural features, providing lipid identification often limited to the species (i.e., lipid class and sum composition) and molecular species (i.e., defined acyl chain composition) levels.

Although outside the scope of this review, IM has proven highly effective for lipid analysis and has been reviewed in detail elsewhere (Heiles 2021; Paglia, Smith, and Astarita 2022; Camunas‐Alberca et al. 2023; Dubland 2022). Consequently, several modern MS platforms integrate IM capabilities, allowing for the additional separation of ions based on their size, shape, and charge measured as the ion's collisional cross section in addition to their *m*/*z* ratio. In short, IM MS improves confidence in lipid identification by providing refined differentiation of lipid isomers and isobars, and its utilization is highly encouraged when available.

Figure 5a highlights the GPL features identifiable with conventional MS/MS (i.e., traditional ESI approaches without novel dissociation or derivatization tactics). A common pitfall in many MS‐based lipidomics works is the over annotation of identified lipid species (Giles et al. 2018; Skotland et al. 2024). To avoid over annotation of lipid structure, the structural hierarchy of lipid identification is briefly presented in Figure 5b. Based on current standardizations and recommendations, molecular species level identification is the lowest hierarchical level of identification achieved by most conventional MS‐based approaches (Liebisch et al. 2020). While CID and HRMS can reduce lipid identification ambiguity, subtle structural features are left unresolved, undermining lipid structural diversity. In particular, low energy CID in any capacity alone cannot reveal subtle lipid structural features like the positions and geometries of carbon–carbon double bonds among others, thus limiting the efficacy of conventional mass spectrometric approaches in lipid structural elucidation (Randolph, Blanksby, and McLuckey 2020). With the given example of PC 34:1, MS/MS facilitates the assignment of the phosphocholine head group and fatty acyl chains (Figure 5a). HRMS would provide resolution of PC 34:1 from the isobaric ether PC *O* 35:1 structure, enabling lipid subclass identification. Unfortunately, additional isomers such as those varying in acyl chain regiochemistry (i.e., sn‐position) along with the positions and geometries of unsaturation sites are left unresolved, prohibiting complete structural annotation and unambiguous lipid identification (Figure 5c). Examples of isomeric/isobaric overlaps for the representative case of PC 34:1 are provided in Figure 5c.

Often referred to as deep lipid profiling, recent works have demonstrated the importance of lipid profiling at detailed structural levels (Bonney and Prentice 2021; Zhang et al. 2022). The ability to perform lipid profiling with C=C specificity (i.e., localization of carbon–carbon double bonds in fatty acyl substituents) has facilitated the identification of modified lipid metabolism in several types of cancer and type‐2 diabetes (Xia et al. 2023; Zhang et al. 2019; Cao et al. 2020; Lin et al. 2022). Importantly, these lipid perturbations would have remained unresolved if relying on traditional lipid profiling techniques alone. Consequently, there is a demand for the continued development of isomer‐specific lipid profiling strategies, namely regarding assigning definitive *sn*‐ and C=C position(s). These approaches have been reviewed elsewhere (Randolph, Blanksby, and McLuckey 2020; Heiles 2021; Bonney and Prentice 2021; Ryan and Reid 2016; Xia and Wan 2023; Blanksby and Mitchell 2010). For example, novel gas‐phase dissociation methods such as ozone‐induced dissociation (OzID), ultraviolet photodissociation (UVPD), electron impact excitation of ions from organics (EIEIO), and ion/ion reactions have proven successful for discerning a range of structural features including the localization of acyl chain regiochemistry and unsaturation sites (Betancourt et al. 2017; Chao and McLuckey 2023; Brodbelt, Morrison, and Santos 2020; Hancock et al. 2017; Randolph et al. 2018; Randolph et al. 2022; Michael et al. 2024; Cvačka, Vrkoslav, and Strnad 2023; Bonney et al. 2023; Baba et al. 2018). Additional derivatization strategies in combination with traditional MS/MS have also enabled isomer‐specific lipid profiling (Randolph et al. 2023). Such approaches include the Paternò‐Büchi reaction, lithium adduction, epoxidation, singlet oxygen derivatization, aziridination, and amidation (Esch and Heiles 2018; Ma and Xia 2014; Zhao et al. 2020; Frankfater and Hsu 2021; Zhao et al. 2017; Cao et al. 2018; Feng et al. 2019; Menzel et al. 2023; Wang, Han, and Han 2013; Unsihuay et al. 2021; Hsu, Bohrer, and Turk 1998; Bowden et al. 2011; Hsu and Turk 1999). Although most instrumental‐based approaches require more intensive MS knowledge and handling, derivatization approaches are often easily accessible to the non‐mass spectrometrist and greatly enhance lipidomic data. For example, amidation kits for deep FA profiling are commercially available (AMP+ MaxSpec Kit, Cayman Chemical), and utilization of lithium adduction requires only lithium‐based salts for doping prior to MS analysis. Future work within the lipidomics MS community should seek to make enhanced MS strategies for deep lipid profiling more accessible to biologists.

Till date, glia lipidomics has been limited to conventional lipid profiling, and given the importance of deep lipid profiling, future investigations should seek alternate MS‐based approaches to identify glia lipids with *at least* C=C specificity to reveal new targets in addition to new biomarkers for disease. While techniques like EIEIO, OzID, UVPD, and ion/ion reactions are not commercially available, strategies reliant on lipid derivatization can be readily employed without instrument modifications or significant investments in experimental materials and are applicable to a wide range of lipid classes, offering an accessible route to deep lipidomics. Noting that approaches for deep lipidomics are reviewed elsewhere, and we urge glia experts to implement their usage on targeted lipid species following lipidomic screening investigations.

## 5 Data Analysis and Bioinformatics

### 5.1 Blank Subtraction, Deconvolution, and Normalization

Like many “omics” disciplines, lipidomics generates large amounts of data, tasking experimentalists with the burden of processing, analyzing, and interpreting complex data sets. Following data acquisition, datasets should be first corrected for any background noise or contamination using a data preprocessing step, often referred to as blank subtraction. Blank subtraction ensures that detected signals originate from the biological sample and are not introduced during sample handling, processing, and mass spectrometric analysis. As lipids are universal biomolecules present in a wide array of materials including solvents and glassware, blank subtraction is a necessary step in any lipidomics workflow, especially for those focusing on FAs.

While the blank subtraction process involves calculating the difference in signal levels from individual test and blank samples, the limit of detection (LOD) is defined by a minimum signal that is statistically distinguishable from this noise. In other words, LOD is the lowest amount of an analyte that can be reliably detected but not necessarily quantified with precision. Often, LOD is calculated based on the signal‐to‐noise ratio (S/N) of 3:1, meaning the analyte signal is three times higher than the noise level. However, standard deviation approaches can also be used. While LOD allows for confirmation of presence, while the limit of quantitation (LOQ)—typically calculated by a S/N of 10:1—allows for reliable, precise quantitation. It is imperative that LOD and LOQ are established in any MS‐based lipidomic workflow, as these parameters provide a clear criterion for the inclusion or exclusion of data, ensuring that only reliable signals are utilized in the ensuing data analysis. Neglecting to establish LOD and LOQ can potentially distort results. Specifically, datasets may contain noise or weak signals, increasing the likelihood of reporting false positives (i.e., detecting signals that are not real) or false negatives and the oversight of low‐level lipids.

There are four main steps in blank subtraction that begin in the sample preparation phase. As a first step, blank samples should be prepared, containing all the reagents and solvents used in the experimental workflow but without the biological sample. In other words, blank controls should go through the entire sample handling procedure including lipid extraction and mass spectrometric analysis. Next, prepared blank controls are analyzed in parallel with biological samples, usually randomized between biological sample analysis sets. After data acquisition, blank signals are subtracted from those of biological samples to improve the accuracy of lipid identification.

Following blank subtraction, datasets should then be corrected for isotopic overlaps (Ryan and Reid 2016; Liebisch et al. 2004; Haimi et al. 2006; Höring et al. 2021). Naturally occurring isotopes are present for lipid‐building block elements such as carbon, hydrogen, oxygen, and nitrogen. The presence of these heavy isotopes, namely carbon‐13, can cause peak overlap in resulting mass spectra. For example, these overlaps often happen between lipid monoisotopic ion peaks which contain only the most abundant isotope of each element and peaks from alternate lipid ion structures containing one or more heavy isotopes. Consequently, isotope correction algorithms are crucial to ensure accurate lipid species identification and quantitation. Without isotopic deconvolution, minor lipid species components can be inflated, leading to inaccurate lipid profiling results. Thus, as it is essential to correct for isotopic contributions in datasets, a number of tools are currently available to ensure that the signal attributed to each lipid species reflects its true abundance. Such tools include: XCMS, MZmine, Metaboanalyst, MS‐DIAL, MSIostope, LipidXplorer, and LipidSearch (Tsugawa et al. 2015).

Another necessary step in lipidomics analysis, especially for semi‐quantitative or quantitative approaches, includes normalization. Normalization helps mitigate experimental condition variations like sample preparation, instrumentation, instrument performance, and ionization efficiency. Moreover, normalization can aid in lipidomics data comparisons across samples and studies. Normalization can be achieved in multiple ways. Popular normalization strategies include internal standard normalization, total ion current (TIC) normalization, median normalization, quantile normalization, and normalization to protein content or cell number. Internal standard normalization has a rich history for quantitative approaches yet presents challenges in many lipidomics workflows due to the lack of heavy isotope labeled lipid standards. TIC normalization is widely accessible and simple. Briefly, TIC normalization involves normalizing the intensity of each lipid species to the TIC (the sum of all detected ion intensities) in the sample. For glia lipidomics, protein count or cell number normalization can be highly beneficial, as it can readily accommodate cell/tissue‐based samples and accounts for variations in sample concentration.

### 5.2 Lipid Identification

To identify lipid species, database matching is commonly employed. Popular online lipid databases include LIPID MAPS, LipidSearch, LipidBlast, Human Metabolome Database (HMDB), and MS‐DIAL (Tsugawa et al. 2015; O'Donnell et al. 2019; Fahy et al. 2007; Kind et al. 2014; Kind et al. 2013; Cajka and Fiehn 2017). Database matching can be broadly grouped by input data type, where the user inputs either MS or MS/MS data. Databases like LIPID MAPS and HMDB hinge on full scan MS data, matching observed *m*/*z* values with theoretical *m*/*z* values of experimentally validated and computationally predicted lipid ions. For the best results, database matching utilizing full scan data requires high‐resolution and mass accuracy. To date, LIPID MAPS is the most comprehensive lipid database, and in turn, is the database of choice for most lipidomic investigations. Regardless of database employed, it is important to note that putative structural assignments should reflect experimental possibilities, meaning that either algorithmic or manual validation is necessary to confirm lipid assignments. In other words, user‐defined input factors such as ion type need to reflect only those plausible given the scan polarity (i.e., positive or negative ion mode). For example, if a user inputs a negative ion mode MS1 peak list, the only possible structures for PC ions are adduct ions (e.g., chloride or acetate) and others should be excluded.

The second type of database matching utilizes MS/MS data to interpret observed fragmentation patterns and assign lipid structure. LipidBlast is an open‐source software and online database for automated MS/MS spectral interpretation (Kind et al. 2014; Kind et al. 2013; Cajka and Fiehn 2017). Notable benefits of LipidBlast include MS platform independence, as it is capable of handling MS/MS data acquired from both high‐ and low‐resolution mass spectrometers. To date, few tools are available to autonomously annotate MRM‐based lipidomic datasets. Skyline is an open‐source software for targeted metabolomics, including MRM‐based lipidomics, that allows users to build custom MRM and full scan quantitative methods, streamlining quantitative workflows (Peng and Ahrends 2016). However, statistical analysis and visualization of MRM results are not supported by Skyline, though tools like R and MetaboAnalyst can help interpret the resulting exported, processed datasets to derive biological insights.

**FIGURE 6:**
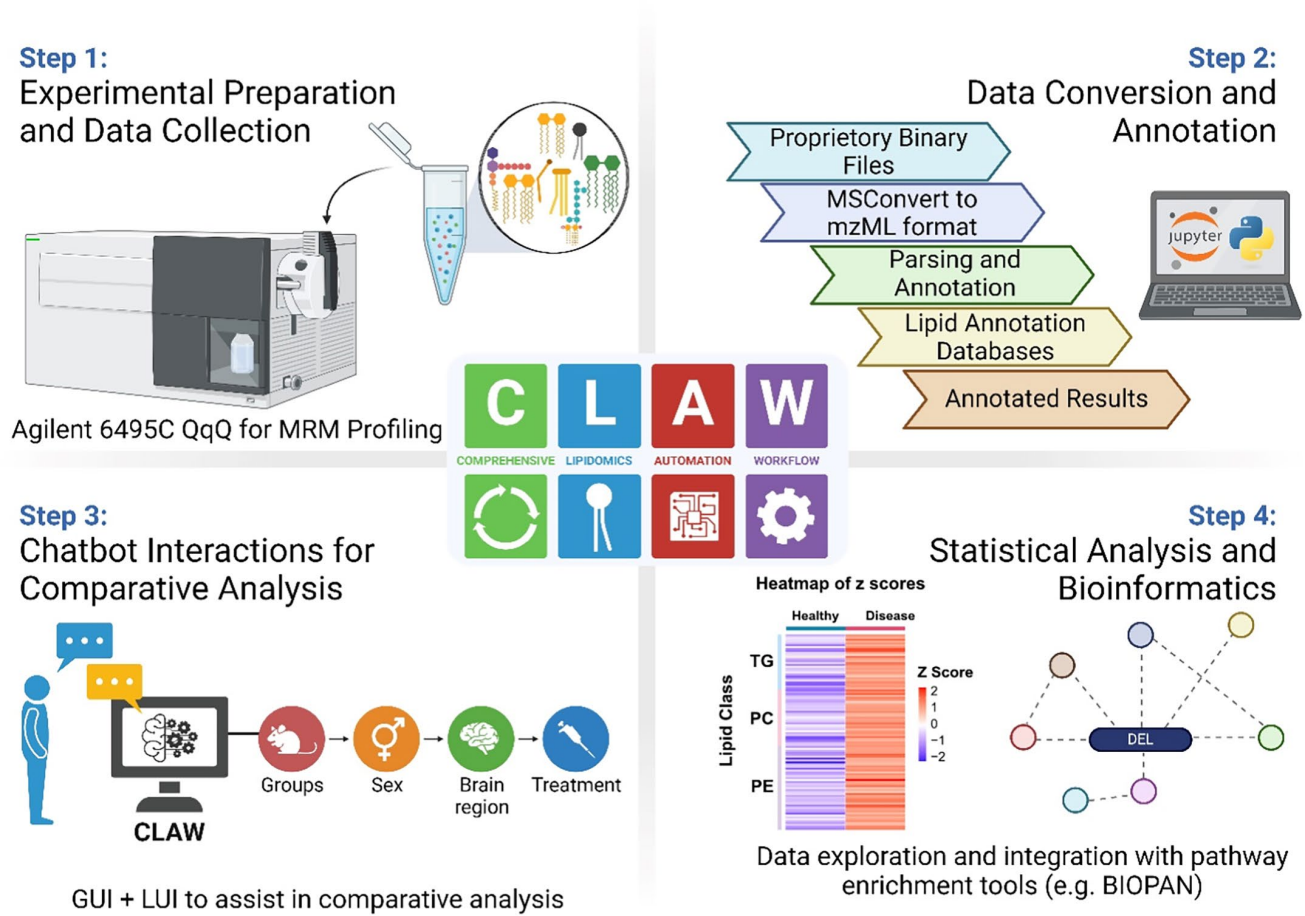
Lipidomics workflow using CLAW. CLAW assists in all stages of lipidomics analysis, including worklist generation, comparative statistical analysis, and integration with pathway enrichment tools like BIOPAN (Gaud et al. 2021). Graphics showing ways to visualize lipidomics data generated using pseudo data.

Noting the lack of tools for MRM lipidomic workflows, Beveridge et al. developed the first end‐to‐end integrated and automated MRM lipidomics platform, referred to as “Comprehensive Lipidomic Automation Workflow” (CLAW) (Beveridge et al. 2024). Notably, CLAW achieves autonomous detailed lipid identification with structural detail (i.e., C=C localization) in complex biological samples while also providing users with streamlined lipidomics workflows, data analysis pipelines, and experimental design aids like worklist generation. By automating processes traditionally done by‐hand, CLAW significantly reduces analysis time, boosts throughput, and provides standardization to data analysis. Finally, to increase usability of the CLAW platform, a language user interface (LUI) and custom artificial intelligence (AI) agents were introduced to assist users in proper data collection, annotation, and interpretation based on large language models (LLMs) via a chatbot‐style interaction (Figure 6). To demonstrate the versatility of CLAW platform, Beveridge et al. reported lipidomics results by using traditional and ozone ESI MRM‐profiling. Here, lipid profiles from multiple complex biological sample types, including canola oil samples and LD extracts from mouse brain were successfully defined and differentiated. In the latter example, roughly 1500 individual lipid species were profiled in LDs isolated from specific brain regions of AD‐model and aged‐matched wild type mice using traditional MRM‐based lipidomics strategies. Interestingly, the data show clear lipidome distinctions among LDs obtained from aged and AD‐diseased brains, indicating the LDs related to aging and AD are not the same. Furthermore, distinct lipid signatures for the LDs isolated from the hippocampus, cortex, cerebellum, and diencephalon regions were observed, revealing region‐specific lipid alterations in both AD and aging.

### 5.3 Statistical Analysis and Data Visualization

Statistical analysis is a crucial step in lipidomic data processing, allowing for the identification of statistically significant or differentially expressed lipids (DELs) along with reductions in data dimensionality. Although outside the scope of this tutorial, statistical analysis methods for lipidomics, including general principles, best practices, and uses, are reviewed elsewhere (Chen, Li, and Xu 2022; Checa, Bedia, and Jaumot 2015; Ni et al. 2023). In short, proper statistical analysis enables complex dataset interpretation so that perturbations in lipid profiles can be discerned, unveiling vital biological and metabolic insights. As mentioned above, R and MetaboAnalyst are popular tools for statistical analysis, though CLAW has recently emerged as a promising platform. Data file type is an important consideration prior to conducting statistical analysis. For example, tools like MetaboAnalyst and CLAW can accept .mzML files but not proprietary instrument data file types. ProteoWizard's MSConvert (https://proteowizard.sourceforge.io/download.html) can be used to convert data files produced by commercial instruments, such as Thermo's RAW or Agilent's .d files, into .mzML format, aiding in statistical analysis. This conversion is typically the first step in a computational pipeline, ensuring the data are in a usable format.

In general, scientific data analysis tools require some level of programming experience to properly process relevant data, posing significant hurdles for many experimentalists. For this reason, user‐friendly tools like CLAW have been developed (Figure 6). CLAW's LUI addresses this problem by assisting the user with proper data analysis and visualization. In short, the LUI functions like a “co‐scientist,” performing user‐defined queries and clarifying specifics regarding data analysis in an interactive manner. This enables users to issue commands via chat‐based input, guiding the LUI agent to filter data for comparative analysis, and perform complex data comparisons in an interactive manner. Briefly, CLAW incorporates both the LUI and custom AI agents which enable users to interact with various components of the workflow ranging from experimental design to data processing. The LUI incorporated into CLAW utilizes a chatbot style interface known as ChatGPT. Incorporation of the LUI can provide text‐based assistance to a user by interacting with tailor‐made AI agents to perform functions. In brief, each AI agent uses the LLM to dynamically process user text, interpret relevant instructions, and select from a predefined set of tools to fulfill tasks. To ensure the user's final answer is achieved, the AI agents repeatedly cycle through a thought‐action‐observation process based on user input and the selected tool output based on the provided instructional prompt. To our knowledge, this represents the first use of an LLM to aid in lipidomics profiling, establishing a novel, robust and comprehensive approach for data analysis.

Across lipidomics, principal component analysis (PCA) is usually one of the first explorative tests conducted to determine variation in samples. In brief, PCA reduces data dimensionality, transforming complex datasets into simpler representations to show variation across all lipids in different samples along each principal component. Consequently, PCA aids in pattern identification, allowing for quick visualizations of data variance among sample groups (e.g., disease vs. healthy controls). Overall lipid abundance can be examined using stacked bar plots or pie charts to provide information about sample composition. Heatmaps are also great for examining trends and variance in sample groups; they can either display the scaled lipid signal intensity or fold change values, depending on the purpose of the analysis. Fold change is a frequently used measure to express changes in lipid levels in response to experimental or biological conditions. Fold change can be calculated using statistical packages such as EdgeR (Robinson, McCarthy, and Smyth 2010). The EdgeR GLM model is particularly suitable for comparative lipidomics analysis due to the negative binomial distribution of the ion count data. This model allows for accurate estimation of fold changes and identification of DELs between groups, accounting for the variability and dispersion inherent in lipidomics data (Benjamini and Hochberg 1995). Visualization methods that employ fold change are crucial for emphasizing disparities in lipid abundances among distinct experimental settings or groups. Ridgeplots, heatmaps, and volcano plots can be beneficial to visualize fold change(s) in global lipid profiles as they display the density distribution of lipids in relation to their fold changes.

Most lipidomic statistical analysis aims at identifying DELs. To summarize, DELs are lipid species with abundances that significantly differ between two or more experimental conditions or groups (e.g., healthy vs. diseased, treated vs. untreated, etc.). For example, DEL identification is a crucial aspect of lipidomics data analysis and interpretation, as DELs can provide insight into the implications of metabolic alterations in disease progression and treatment. DEL identification can be achieved using statistical tests like *t* tests and analysis of variance. Correction factors like false discovery rate (FDR) are advantageous to ensure the accuracy of identified DEL species, as FDR controls the expected proportion of false positives. Therefore, the implementation of FDR correction will enhance the caliber and precision of lipidomic data, facilitating more precise biological interpretations and discoveries. Following FDR correction and DEL discovery, plots like volcano plots, scatterplots, or box plots can be advantageous for visualization. Sample R codes for data analysis and visualization can be found at our Github repository (https://github.com/chopralab). Additionally, the use of colors in any data visualization should be accessible to viewers with color vision deficiency (CVD). Online resources such as IWantHue (iWantHue [medialab.github.io] and Coolors; https://coolors.co/) can generate custom CVD palettes. There is also a large collection CVD‐safe palettes in multiple R packages, including as ggsci, vridis, and RColorBrewer. R packages like colorblindr, dichromat, and colorspace can also be used to simulate CVD to ensure accessibility (Zeileis et al. 2020).

### 5.4 Pathway Analysis

Enrichment and pathway analyses identify relevant biological pathways which potentially underly the observed lipidomic changes. Lipidomics data are mapped to known biochemical processes in databases like KEGG or Reactome using specialized software or web‐based tools (Kanehisa and Goto 2000; Milacic et al. 2024). A comprehensive list of lipidomic pathway analysis tool can be found in recent reviews (Chen, Li, and Xu 2022; Ni et al. 2023). Enrichment analyses consider simultaneous changes in multiple lipid species or classes, translating into higher confidence in significant discoveries. Some of our favorite pathway enrichment analyses platforms include LipidSig 2.0, LIPEA, and BIOPAN which are all web‐based with detailed, user‐friendly instructions (Gaud et al. 2021; Liu et al. 2024; Acevedo et al. 2018). Both LipidSig2.0 and BIOPAN provide lipid‐related genes involved in the predicted pathways which are very useful for correlating between lipidomics and existing transcriptomics or proteomics datasets. However, current tools for enrichment analyses share several limitations, such as the requirement for data to be in a specific format, limited number of lipid species/classes in the library, and lack of integration with other omics data. While platforms like LIPEA and LipidSig offer valuable insight, they do have major limitations that impact their reliability. Specifically, these platforms only focus on changes in major lipid classes without considering more nuanced, yet important lipid characteristics, such as fatty acyl chain length and degree of unsaturation. This is a significant drawback because variations in chain length and saturation of lipid molecules have been shown to be crucial indicators in various pathologies and can greatly influence biological functions and disease mechanisms. BIOPAN has improved upon other tools by matching lipids based on acyl chain length and degree of unsaturation. Future pathway enrichment tools should address these limitations as well as provide better user‐friendly tools for data manipulation.

## 6 Conclusions and Outlook

Largely attributed to significant advances in MS instrumentation, lipidomics has been propelled as an irreplaceable pillar in omics‐based disciplines. Once considered a subdiscipline of metabolomics, lipidomics now serves as an independent field, providing a snapshot of cellular state along with unique insights into metabolic alterations associated with a diverse range of conditions. It is well known that glial cells serve essential functions in the CNS. Furthermore, as lipids also have specialized functions in the CNS, lipids are vital for the health and functioning of glial cells. Consequently, understanding perturbations to the glia lipidome in response to stressors like neurodegeneration and inflammation can provide vital insights into disease mechanisms, further enabling the discovery and development of novel therapeutic approaches to combat disease progression.

In this tutorial, we aimed to provide readers with an introductory guide to MS‐based glia lipidomics, briefly reviewing the basic steps of typical lipidomic workflows, ranging from sample collection and preparation down to data interpretation. Special considerations around lipid extraction and sample preparation should be noted to ensure the accuracy and quality of lipidomic data. Additionally, we urge the community to adhere to current lipidomics standardization initiatives regarding lipid nomenclature, structural annotation, method development, and data transparency to ensure comparisons across labs, platforms, and data sets. The characteristics of multiple MS‐based approaches were also detailed to help guide users based on their individual needs, in an effort to clarify the capabilities of various mass spectrometric platforms and output lipidomic results.

In short, due to its ease of use, speed, and efficacy, we advocate for the utilization of shotgun MRM‐profiling as a first step in any glia lipidomic investigation. With extensive experience in glia lipidome MRM profiling, we summarized multiple notable studies by our lab and others, along with their larger biological implications, highlighting the importance of glia lipidomics. For completeness, results of MRM‐screening should be confirmed with hyphenated MS‐based methods employing high‐performance separation strategies like LC or IM. Finally, isomer‐specific MS/MS approaches that entail novel dissociation and/or chemical derivatization strategies were presented to highlight the future direction of MS‐based lipidomics, as these methodologies enable deep lipid profiling. To date, deep lipid profiling has been unaddressed in glia lipidomics, presenting a significant knowledge gap in the field. By achieving complete lipid structure elucidation, the roles of specific lipid species can be better understood and leveraged to combat a broad range of neurological conditions, including neurodegenerative diseases like AD.

## Author Contributions

Conceptualization: C.E.R. and G.C. Writing – original draft, review and editing: C.E.R., K.A.W., R.Y., C.B., P.M., and G.C. Supervision and funding acquisition: G.C.

## Conflicts of Interest

G.C. is the Director of the Merck‐Purdue Center funded by Merck Sharp & Dohme, a subsidiary of Merck and the co‐founder of Meditati Inc. and BrainGnosis Inc. The remaining authors declare no conflicts of interest.

## Data Availability

The authors have nothing to report.
